# Impact of renin-angiotensin system inhibitors on the survival of patients with rectal cancer

**DOI:** 10.1186/s12885-022-09919-0

**Published:** 2022-07-25

**Authors:** Marcin Zeman, Władysław Skałba, Agata Małgorzata Wilk, Alexander Jorge Cortez, Adam Maciejewski, Agnieszka Czarniecka

**Affiliations:** 1 Gliwice Branch, The Oncologic and Reconstructive Surgery Clinic, Maria Skłodowska-Curie National Research Institute of Oncology, Wybrzeże Armii Krajowej 15, 44-102 Gliwice, Poland; 2 Department of Biostatistics and Bioinformatics, Gliwice Branch, Maria Skłodowska-Curie National Research Institute of Oncology, Wybrzeże Armii Krajowej 15, 44-102 Gliwice, Poland; 3 Department of Systems Biology and Engineering, Silesian University of Technology, Akademicka 16, 44-100 Gliwice, Poland

**Keywords:** Rectal cancer, Renin-angiotensin system inhibitors, Angiotensin-converting enzyme inhibitors, ACEI, Angiotensin receptor blockers, ARB, Arterial hypertension

## Abstract

**Background:**

Renin-angiotensin system inhibitors (RASIs) are widely used in the treatment of hypertension. However, their impact on the outcome of the combined treatment of rectal cancer is poorly understood. The aim of this study was to assess the effect of RASIs on the survival of rectal cancer patients with associated hypertension after neoadjuvant treatment and radical resection.

**Methods:**

Between 2008 and 2016, 242 radical (R0) rectal resections for cancer were performed after neoadjuvant treatment in patients with associated hypertension. At the time of treatment, 158 patients were on RASIs, including 35 angiotensin-receptor antagonists (ARB) users and 123 angiotensin-converting enzyme inhibitors (ACEI) users. Eighty-four patients were on drugs other than RASIs (non-RASI users). The survival analysis was performed using the Kaplan–Meier estimator with the log-rank test and the Cox proportional hazards model.

**Results:**

The log-rank test showed a significantly worse overall survival (OS) in the group of ACEI users compared to ARB users (*p* = 0.009) and non-RASI users (*p* = 0.013). Disease-free survival (DFS) was better in the group of ARB users compared to ACEI users. However, the difference was not statistically significant (*p* = 0.064). The Multivariate Cox analysis showed a significant beneficial effect of ARBs on OS (HR: 0.326, 95% CI: 0.147–0.724, *p* = 0.006) and ARBs on DFS (HR: 0.339, 95% CI: 0.135–0.850, *p* = 0.021) compared to ACEIs. Other factors affecting OS included age (HR: 1.044, 95% CI: 1.016–1.073, *p* = 0.002), regional lymph node metastasis (ypN +) (HR: 2.157, 95% CI: 1.395–3.334, *p* = 0.001) and perineural invasion (PNI) (HR: 3.864, 95% CI: 1.799–8.301, *p* = 0.001). Additional factors affecting DFS included ypN + (HR: 2.310, 95% CI: 1.374–3.883, *p* = 0.002) and PNI (HR: 4.351, 95% CI: 1.584–11.954, *p* = 0.004).

**Conclusions:**

The use of ARBs instead of ACEIs may improve the outcome of the combined therapy for rectal cancer patients with associated hypertension.

**Supplementary Information:**

The online version contains supplementary material available at 10.1186/s12885-022-09919-0.

## Background

Hypertension is a common comorbidity in patients with colorectal cancer [1]. In addition, it was shown that patients with hypertension could have an increased risk of developing colorectal cancer [2]. The circulatory renin-angiotensin system (RAS) is a regulator of sodium and water homeostasis. It is one of the phylogenetically oldest endocrine systems of vertebrates [3]. In kidney cells, prorenin is converted to renin, which is secreted into the circulation. Renin causes the conversion of angiotensinogen produced in the liver to angiotensin I, which is then converted to angiotensin II (AngII) by angiotensin-converting enzyme (ACE). AngII can directly act on vessel walls causing their contraction, and it stimulates the adrenal cortex to secrete aldosterone. Furthermore, the presence of tissue RAS (tRAS) was demonstrated. It plays an important role in the pathogenesis of cardiovascular, inflammatory, autoimmune, and neoplastic diseases [4]. The presence of tRAS was demonstrated within normal and tumor tissues, including the tumor microenvironment [5, 6]. It has the impact on tumor cells via two mechanisms, i.e. via the AngII type 1 receptor (AT1R) and the AngII type 2 receptor (AT2R). AT1R activation leads to the activation of pro-inflammatory and pro-angiogenic pathways, while AT2R activation has the opposite effect (anti-inflammatory, anti-proliferative and anti-angiogenic) [7].

RAS inhibitors (RASIs), which include angiotensin-converting enzyme inhibitors (ACEIs) and angiotensin receptor antagonists (ARBs), are widely used in the treatment of arterial hypertension. Although both groups of drugs block the RAS and tRAS, their mechanism of action is different. ACEIs inhibit AngII production via ACE inhibition. However, it was shown that despite ACE inhibition, the pro-tumor pathway via AT1R could still be activated by an ACE-independent pathway by chymase, which is an enzyme that is activated under conditions of local inflammation [8]. In addition, ACEIs influence the kallikrein-kinin system (KKS) by inhibiting the catabolism of pro-invasive kinins to inactive metabolites. However, the above effects are not reported for ARBs, which block the action of AngII by selective antagonism of the AT1R, nor do they show an effect on KKS [9].

Population-based studies showed that RASIs could reduce the prevalence of colorectal cancer. However, their impact on the long-term outcomes of colorectal cancer has been poorly understood [10]. In many studies, the influence of both groups of these drugs on the results of cancer treatment is analyzed jointly. However, it seems that due to the different mechanisms of action, these groups should be assessed separately.

## Methods

### Aim of the study

To assess the effect of RASIs on overall survival (OS) and disease-free survival (DFS) of rectal cancer patients without synchronous distant metastases with associated hypertension after neoadjuvant treatment and radical resection.

### Patients

Between 2008 and 2016, 242 radical (R0) rectal resections for cancer were performed at our center after neoadjuvant treatment in patients without distant metastases with associated hypertension. The enrolment procedure is shown in the diagram [see Additional file 1]. To avoid including patients with synchronous microdissemination in the analysis, metastases clinically detected within 3 months postoperatively were considered synchronous metastases. The severity of the associated diseases was assessed using the Charlson comorbidity index (CCI) [11].

### Procedures

All patients received neoadjuvant treatment, i.e., radiotherapy (RT) at a total dose of 25–42 Gy or chemoradiotherapy (CRT) at a dose of 42–54 Gy combined with one or two cycles of 5-fluorouracil-based chemotherapy. All procedures were performed by colorectal surgeons or under their direct supervision. Laparotomy with total mesorectal excision was performed. Postoperative complications were assessed using the Clavien-Dindo scale. Tumor regression grade (TRG) was based on the assessment of the degree of fibrosis compared to the residual tumor tissue and ranged from 0 to 3, i.e., 0 (complete response), 1 (< 10% residual tumor), 2 (10–50%) and 3 (> 50%). During the analyzed period, adjuvant chemotherapy was based on 5-fluorouracil. The characteristics of the study group are shown in Table 1. After the end of treatment, all patients were under continuous follow-up in our center.

**Table 1:** Patient characteristics

		Total number of patients (*n* = 242)	ACEI *n* = 123	ARB *n* = 35	p	RASI *n* = 158	Non-RASI *n* = 84	p
Age	median (IQR)	68 (62–73)	69 (63–74)	67(60.5–69.5)	0.074	68 (62–74)	67 (61–72)	0.460
Sex	Females	109 (45.04%)	53 (43.09%)	21 (60.00%)	0.087	74 (46.84%)	35 (41.67%)	0.498
	Males	133 (54.96%)	70 (56.91%)	14 (40.00%)		84 (53.16%)	49 (58.33%)	
BMI	median (IQR)	26.8 (24.6–30.475)	26.8 (24.75–29.82)	28.4 (25.75–30.95)	0.083	27(24.92–30.575)	26.4 (24.575–30.4)	0.420
CAD	Yes	52 (21.49%)	25 (20.33%)	5 (14.29%)	0.476	30 (18.99%)	22 (26.19%)	0.250
	No	190 (78.51%)	98 (79.67%)	30 (85.71%)		128 (81.01%)	62 (73.81%)	
DM	Yes	70 (28.93%)	35 (28.46%)	12 (34.29%)	0.533	47 (29.75%)	23 (27.38%)	0.767
	No	172 (71.07%)	88 (71.54%)	23 (65.71%)		111 (70.25%)	61 (72.62%)	
CKD	Yes	6 (2.48%)	2 (1.63%)	1 (2.86%)	0.531	3 (3.57%)	3 (1.90%)	0.421
	No	236 (97.52%)	121 (98.37%)	34 (97.14%)		81 (96.43%)	155 (98.10%)	
CCI	0–2	123 (50.83)	61 (49.59%)	18 (51.43%)	1	79 (50.00%)	44 (52.38%)	0.787
	> 2	119 (49.17)	62 (50.41%)	17 (48.57%)		79 (50.00%)	40 (47.62%)	
cTNM Stage	2	73 (30.17)	37 (30.08%)	16 (45.71%)	0.105	53 (33.54%)	20 (23.81%)	0.141
	3	169 (69.83)	86 (69.92%)	19 (54.29%)		105 (66.46%)	64 (76.19%)	
Distance to the anal verge	< = 5 cm	138 (57.02%)	76 (61.79%)	17 (48.57%)	0.350	93 (58.86%)	45 (53.57%)	0.663
	6–10 cm	69 (28.51%)	32 (26.02%)	12 (34.29%)		44 (27.85%)	25 (29.76%)	
	11–15 cm	35 (14.46%)	15 (12.20%)	6 (17.14%)		21 (13.29%)	14 (16.67%)	
Neo-adjuvant	RT	178 (73.55%)	93 (75.61%)	25 (71.43%)	0.661	118 (74.68%)	60 (71.43%)	0.647
	CRT	64 (26.45%)	30 (24.39%)	10 (28.57%)		40 (25.32%)	24 (28.57%)	
Surgery	AR	135 (55.79%)	67 (54.47%)	19 (54.29%)	1	86 (54.43%)	49 (58.33%)	0.237
	APR	96 (39.67%)	52 (42.28%)	15 (42.86%)		67 (42.41%)	29 (34.52%)	
	Hartm	11 (4.55%)	4 (3.25%)	1 (2.86%)		5 (3.16%)	6 (7.14%)	
Clavien	0–2	204 (84.30%)	107 (86.99%)	33 (94.29%)	0.366	140 (88.61%)	64 (76.19%)	0.015
	> 2	38 (15.70%)	16 (13.01%)	2 (5.71%)		18 (11.39%)	20 (23.81%)	
ypT	0–1	25 (10.33%)	9 (7.32%)	4 (11.43%)	0.454	13 (8.23%)	12 (14.29%)	0.143
	2	86 (35.54%)	51 (41.46%)	11 (31.43%)		62 (39.24%)	24 (28.57%)	
	3–4	131 (54.13%)	63 (51.22%)	20 (57.14%)		83 (52.53%)	48 (57.14%)	
ypN	positive	91 (37.60%)	43 (34.96%)	14 (40.00%)	0.690	57 (36.08%)	34 (40.48%)	0.577
	negative	151 (62.40%)	80 (65.04%)	21 (60.00%)		101 (63.92%)	50 (59.52%)	
LNY	median (IQR)	11.5 (8–16)	12 (8–16)	11 (7.5–15.5)	0.620	11.5 (8–16)	11.5 (8–16)	0.666
TRG	0–1	88 (36.36%)	40 (32.52%)	9 (25.71%)	0.537	49 (31.01%)	39 (46.43%)	0.024
	2–3	154 (63.64%)	83 (67.48%)	26 (74.29%)		109 (68.99%)	45 (53.57%)	
LVI	Yes	8 (3.31%)	6 (2.50%)	0 (0.00%)	0.340	6 (3.80%)	2 (2.38%)	0.717
	No	234 (96.69%)	117 (97.50%)	35 (100%)		152 (96.20%)	82 (97.62%)	
PNI	Yes	9 (3.72%)	4 (3.25%)	1 (2.86%)	1	5 (3.16%)	4 (4.76%)	0.503
	No	233 (96.28%)	119 (96.75%)	34 (97.14%)		153 (96.84%)	80 (95.24%)	
Adjuvant CT	Yes	81 (33.47%)	41 (33.33%)	12 (34.29%)	1	53 (33.54%)	28 (33.33%)	1
	No	161 (66.53%)	82 (66.67%)	23 (65.71%)		105 (66.46%)	56 (66.67%)	
Adjuvant CT > 3 cycles	Yes	73 (30.17%)	35 (28.46%)	13 (37.14%)	0.405	48 (30.38%)	25 (29.76%)	1
	No	169 (69.83%)	88 (71.54%)	22 (62.86%)		110 (69.62%)	59 (70.24%)	
CT cycles	Median (IQR)	1 (0–4.75)	1 (0–4)	0 (0–6)	0.711	0.5 (0–4)	1 (0–5.25)	0.480

*SD* Standard deviation, *ACEI* Angiotensin-converting enzyme inhibitors, *ARB* Angiotensin receptor blockers, *RASI* Renin-angiotensin system inhibitors, *IQR* Interquartile range, *BMI* Body mass index, *CAD* Coronary artery disease, *DM* Diabetes mellitus, *CKD* Chronic kidney disease, *CCI* Charlson Comorbidity Index, *RT* Radiotherapy, *CRT* Chemoradiotherapy, *AR* Anterior resection, *APR* Abdominoperineal resection, *Hartm* Hartmann’s procedure, *Clavien* Severity of postoperative complications according to the Clavien-Dindo classification, *yG* Tumor grade, *LNY* Lymph node yield, *TRG* Tumor regression grade, *LVI* Lymphovascular invasion, *PNI* Perineural invasion, *CT* Chemotherapy

### Variables

The following potential risk factors were considered in the survival analysis: age, sex, body mass index (BMI), medication status at the time of surgery, tumor location in the rectum, neoadjuvant treatment (RT or CRT), cancer stage before treatment, type of surgery, occurrence of postoperative complications, tumor invasion depth (ypT), nodal staging (ypN), lymph vessel invasion (LVI), perineural invasion (PNI), TRG, lymph node yield (LNY), adjuvant chemotherapy, concomitant disease status according to the CCI and separately diabetes mellitus (DM) and coronary artery disease (CAD). Chronic kidney disease (CKD) was not included in the analysis due to a small number of patients with this condition.

### Statistical methods

Categorical variables were summarized as frequencies and percentages, and continuous variables were shown as median values with interquartile ranges (25% to 75%, IQR 25–75) unless otherwise stated. Pairwise comparisons between patient subgroups were performed by the Fisher exact test for categorical variables, and the odds ratio (OR) was calculated. For continuous variables, comparisons between two groups were determined using the Wilcoxon rank sum test.

OS was defined as the time from surgery until death, or the last known date alive. DFS was calculated from the time of surgery to the date of the last follow-up without the development of local or distant recurrence. The survival analysis was performed using the survival package (v. 3.2–7) [12] and the glmnet package (v. 4.1–1) [13]. Visualizations were prepared with the survminer package (v. 0.4.8) [14]. Survival curves were plotted with the Kaplan–Meier method and compared using the log-rank test (the Mantel–Haenszel test). Univariate and multivariate analyses with the survival endpoint were investigated by the Cox proportional-hazards model, verifying the proportional hazard assumption with Schoenfeld residuals. Significant risk factors were selected by applying several methods, i.e., preselection with the univariate Cox analysis (variables with *p*-value < 0.200 were included in the multivariate analysis), recursive elimination based on the Akaike information criterion (AIC), and the least absolute shrinkage and selection operator (LASSO) [15]. The complete report from Cox proportional-hazards model regression analyses is given in Additional file 2.

All analyses were performed using the R environment for statistical computing version 4.0.2 “Taking off Again” released on June 22, 2020 (R Foundation for Statistical Computing, Vienna, Austria, http://www.r-project.org). A two-sided *p*-value < 0.05 was considered statistically significant.

## Results

At the time of treatment, 158 patients were on RASIs, including 35 ARB users and 123 ACEI users. Eighty-four patients were on drugs other than RASIs (non-RASI users). No significant differences between ARB and ACEI users were found in the frequency of use of other drug groups. Non-RASI users significantly more frequently used beta blockers compared to RASI users (*p* = 0.001, OR = 2.619). The drugs used in each group are shown in Table 2. The use of RASIs is shown in Table 3. We found a higher prevalence of complications > grade II (Clavien–Dindo Classification) (*p* = 0.015, OR = 2.421) and better response (TRG 0–1) to neoadjuvant treatment (*p* = 0.024, OR = 1.923) in the group of non-RASI users compared to RASI users.

**Table 2:** Drugs used in the study groups

		ACEI *n* = 123 n (%)	ARB *n* = 35 n (%)	p	RASI *n* = 158 n (%)	Non-RASI *n* = 84 n (%)	p
Alpha blockers	Yes	10 (8.13%)	3 (8.57%)	1	13 (8.23%)	6 (7.14%)	1
	No	113 (91.87%)	32 (91.43%)		145 (91.77%)	78 (92.86%)	
Beta blockers	Yes	64 (52.03%)	13 (37.14%)	0.130	77 (48.73%)	60 (71.43%)	0.001
	No	59 (47.97%)	22 (62.86%)		81 (51.27%)	24 (28.57%)	
Calcium channel blockers	Yes	29 (23.58%)	7 (20.00%)	0.820	36 (22.78%)	19 (22.62%)	1
	No	94 (76.42%)	28 (80.00%)		122 (77.22%)	65 (77.38%)	
Diuretics	Yes	35 (28.46%)	15 (42.86%)	0.148	50 (31.65%)	31 (36.90%)	0.475
	No	88 (71.54%)	20 (57.14%)		108 (68.35%)	53 (63.10%)	
Nitrates	Yes	13 (10.57%)	1 (2.86%)	0.308	14 (8.86%)	9 (10.71%)	0.650
	No	110 (89.43%)	34 (97.14%)		144 (91.14%)	75 (89.29%)	
Statins	Yes	6 (4.88%)	2 (5.71%)	1	8 (5.06%)	2 (2.38%)	0.501
	No	117 (95.12%)	33 (94.29%)		150 (94.94%)	82 (97.62%)	

*ACEI* Angiotensin-converting enzyme inhibitors, *ARB* Angiotensin receptor blockers, *RASI* Renin-angiotensin system inhibitors

**Table 3:** RASIs (ACEIs and ARBs) used in the study groups

Group	Drug	n(%)
ACEI	ramipril	49 (39.8)
	enalapril	28 (22.8)
	perindopril	16 (13.0)
	cilazapril	8 (6.5)
	lisinopril	8 (6.5)
	ramipril	5 (4.1)
	quinapril	4 (3.3)
	trandolapril	3 (2.4)
	imidapril	1 (0.8)
	zofenopril	1 (0.8)
ARB	losartan	18 (51.4)
	valsartan	11 (31.4)
	telmisartan	6 (17.2)

*ACEI* Angiotensin-converting enzyme inhibitors, *ARB* Angiotensin receptor blockers, *RASI* Renin-angiotensin system inhibitors

**Fig. 1:**
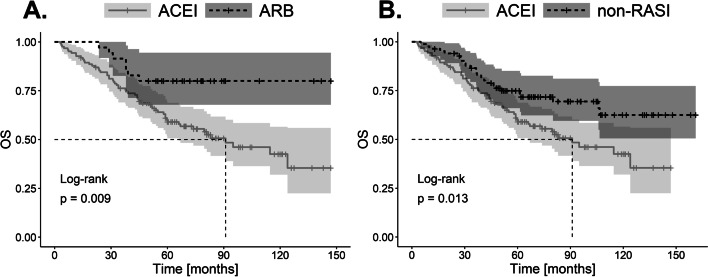
Kaplan–Meier plots of overall survival (OS) for the comparison of patient groups (**A**) ARBs vs. ACEIs and (**B**) ACEIs vs. non-RASIs

We found a significantly worse OS (*p* = 0.009) in the ACEI-treated group (the log-rank test) compared to ARB-treated patients and non-RASI users (*p* = 0.013) (Fig. 1). However, no significant difference in OS (*p* = 0.293) was found when ARB users were compared to non-RASI users (*p* = 0.293) [see Additional file 3A].

**Fig. 2:**
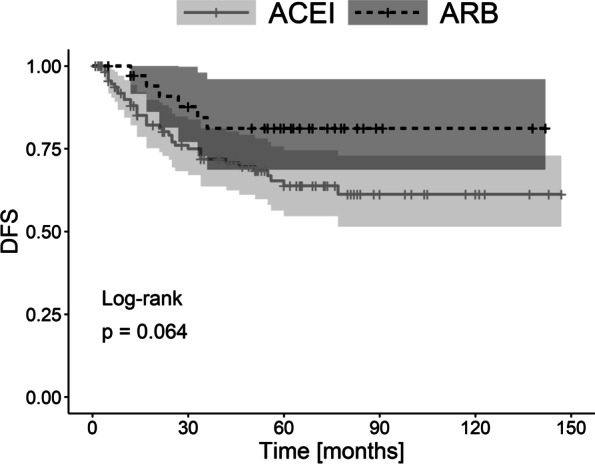
The Kaplan–Meier plot of disease-free survival (DFS) for the comparison of patient groups (ARBs vs. ACEIs)

DFS was better in the group of ARB users compared to ACEI users. However, the difference was not statistically significant (*p* = 0.064) (Fig. 2). No difference was found in DFS between ARB users and non-RASI users (*p* = 0.201). Similarly, no difference was reported for DFS when ACEI users were compared to non-RASI users (*p* = 0.429) [see Additional file 3B].

**Fig. 3:**
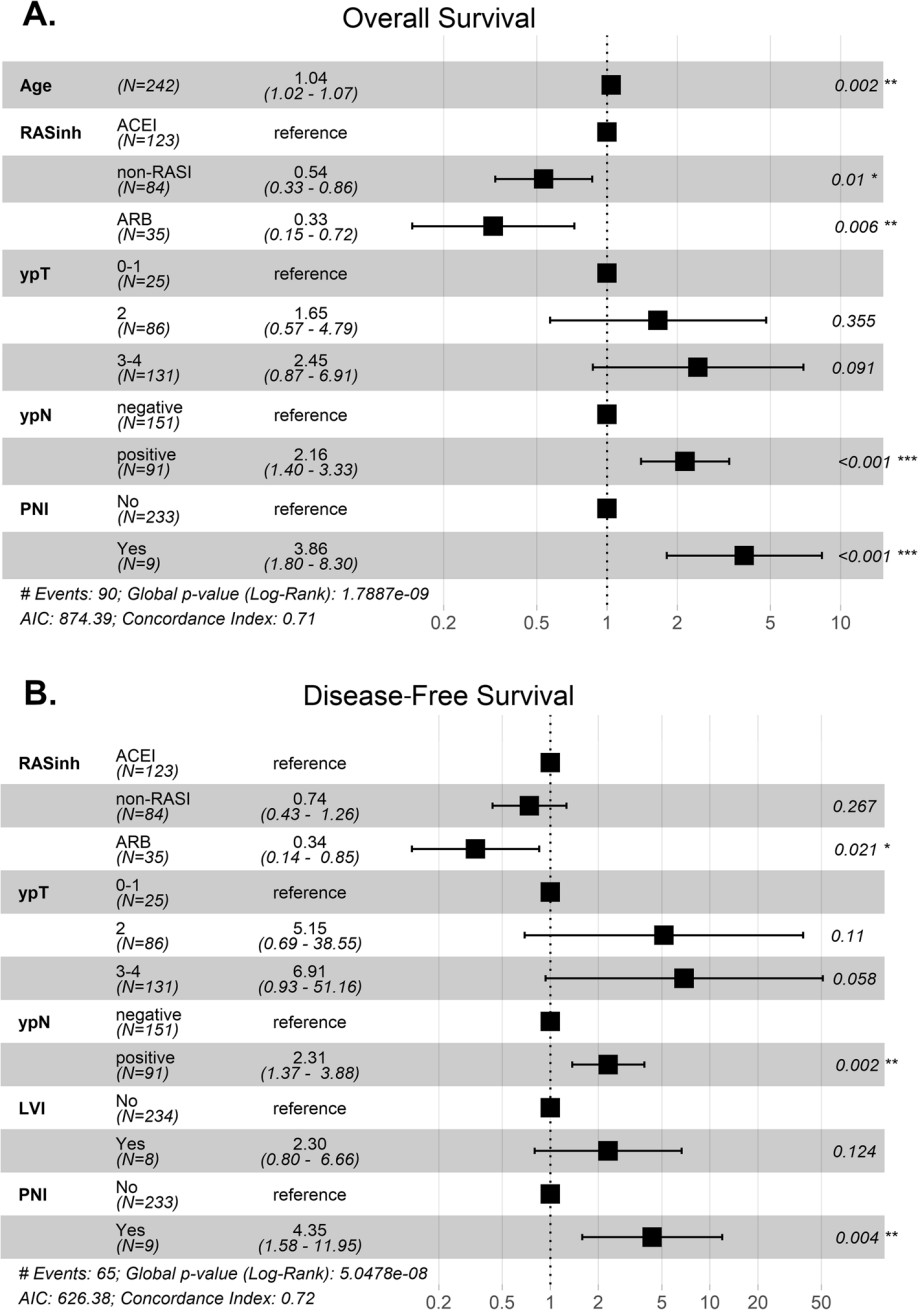
The forest plots of hazard ratio results from a reduced multivariate Cox regression model for (**A**) overall survival (OS) and (**B**) disease-free survival (DFS) prognostic factors; * indicates *p* < 0.05, ** *p* < 0.01, *** *p* < 0.001

Univariate and multivariate Cox regression models are shown in Table 4. In the multivariate analysis of OS, adverse risk factors included age (HR: 1.044, 95% CI: 1.016–1.073, *p* = 0.002), ypN + (HR: 2.157, 95% CI: 1.395–3.334, *p* = 0.001) and PNI (HR: 3.864, 95% CI: 1.799–8.301, *p* = 0.001). Compared to ACEI users, a significant beneficial effect was found in the case of non-RASI users (HR: 0.536, 95% CI: 0.333–0.864, *p* = 0.010) and ARB users (HR: 0.326, 95% CI: 0.147–0.724, *p* = 0.006) (Fig. 3A). For DFS, unfavorable factors included ypN + (HR: 2.310, 95% CI: 1.374–3.883, *p* = 0.002) and PNI (HR: 4.351, 95% CI: 1.584–11.954, *p* = 0.004). A significant beneficial effect was demonstrated in ARB users (HR: 0.339, 95% CI: 0.135–0.850, *p* = 0.021) (Fig. 3B). The other analyzed factors did not have a significant influence on survival.

**Table 4:** Multivariate Cox proportional hazards models for OS and DFS

Variables	OS						DFS					
	uHR (95% CI)	*p*	mHR (95% CI)	*P*	mrHR (95% CI)	*p*	uHR (95% CI)	*P*	mHR (95% CI)	*P*	mrHR (95% CI)	*p*
**Age**												
	1.045 (1.017–1.073)	**0.002**	1.044 (1.016–1.074)	**0.002**	1.044 (1.016–1.073)	**0.002**	1.010 (0.980–1.042)	0.515				
**Sex**												
Females	[Reference] 1						[Reference] 1					
Males	1.199 (0.786 – 1.045)	0.399					1.326 (0.804–2.186)	0.269				
**BMI**												
	0.995 (0.950–1.041)	0.816					1.033 (0.983–1.087)	0.201				
**CAD**												
No	[Reference] 1						[Reference] 1					
Yes	1.158 (0.710–1.888)	0.557					0.819 (0.437–1.532)	0.531				
**DM**												
No	[Reference] 1						[Reference] 1					
Yes	1.135 (0.726–1.774)	0.579					0.931 (0.540–1.603)	0.796				
**CCI**												
0–2	[Reference] 1						[Reference] 1					
> 2	1.260 (0.832–1.906)	0.275					0.890 (0.547–1.450)	0.641				
**cTNM Stage**												
2	[Reference] 1						[Reference] 1					
3	1.311 (0.822–2.093)	0.256					1.245 (0.723–2.143)	0.430				
**Distance to the anal verge**												
< = 5 cm	[Reference] 1						[Reference] 1		[Reference] 1			
6–10 cm	0.871 (0.539–1.408)	0.574					0.810 (0.465–1.411)	0.457	0.803 (0.456–1.416)	0.449		
11–15 cm	0.949 (0.495–1.818)	0.874					0.538 (0.228–1.268)	0.157	0.605 (0.249–1.465)	0.265		
**Neoadjuvant**												
RT	[Reference] 1		[Reference] 1				[Reference] 1		[Reference] 1			
CRT	0.573 (0.333–0.985)	**0.044**	0.743 (0.425–1.300)	0.298			0.552 (0.295–1.033)	0.063	0.783 (0.406–1.512)	0.467		
**Surgery**												
AR	[Reference] 1		[Reference] 1				[Reference] 1					
APR	1.455 (0.960–2.205)	0.077	1.398 (0.912–2.144)	0.125			1.332 (0.806–2.201)	0.264				
Hartm	0.635 (0.087–4.637)	0.654	0.547 (0.072–4.153)	0.560			1.688 (0.515–5.527)	0.387				
**Clavien**												
0–2	[Reference] 1						[Reference] 1		[Reference] 1			
> 2	1.411 (0.832–2.394)	0.202					1.645 (0.895–3.022)	0.109	1.584 (0.782–3.205)	0.201		
**ypT**												
0–1	[Reference] 1		[Reference] 1		[Reference] 1		[Reference] 1		[Reference] 1		[Reference] 1	
2	1.943 (0.678–5.567)	0.216	1.810 (0.617–5.308)	0.280	1.653 (0.570–4.793)	0.355	6.396 (0.860–47.556)	0.070	4.951 (0.657–37.345)	0.121	5.154 (0.689–38.551)	0.110
3–4	3.490 (1.268–9.608)	**0.016**	2.587 (0.912–7.342)	0.074	2.449 (0.868–6.909)	0.091	11.507 (1.584–83.595)	**0.016**	6.757 (0.900–50.741)	0.063	6.910 (0.933–51.157)	0.058
**ypN**												
negative	[Reference] 1		[Reference] 1		[Reference] 1		[Reference] 1		[Reference] 1		[Reference] 1	
Positive	2.192 (1.448–3.316)	** < 0.001**	2.090 (1.343–3.251)	**0.001**	2.157 (1.395–3.334)	**0.001**	2.890 (1.769–4.721)	** < 0.001**	2.068 (1.086–3.939)	**0.027**	2.310 (1.374–3.883)	**0.002**
**LNY**												
	1.013 (0.982–1.045)	0.426					1.018 (0.980–1.057)	0.362				
**TRG**												
0–1	[Reference] 1						[Reference] 1		[Reference] 1			
2–3	1.296 (0.832–2.017)	0.251					1.824 (1.059–3.144)	**0.030**	1.339 (0.750–2.391)	0.324		
**LVI**												
No	[Reference] 1		[Reference] 1				[Reference] 1		[Reference] 1		[Reference] 1	
Yes	3.920 (1.795–8.562)	**0.001**	1.038 (0.440–2.447)	0.932			8.975 (3.765–21.397)	** < 0.001**	2.367 (0.771–7.258)	0.132	2.303 (0.796–6.665)	0.124
**PNI**												
No	[Reference] 1		[Reference] 1		[Reference] 1		[Reference] 1		[Reference] 1		[Reference] 1	
Yes	4.912 (2.351–10.260)	** < 0.001**	3.415 (1.519–7.678)	**0.003**	3.864 (1.799–8.301)	**0.001**	7.619 (3.412–17.013)	** < 0.001**	3.134 (1.056–9.297)	**0.040**	4.351 (1.584–11.954)	**0.004**
**Adj CT > 3 cycles**												
No	[Reference] 1						[Reference] 1		[Reference] 1			
Yes	1.284 (0.836–1.972)	0.254					2.167 (1.330–3.529)	**0.002**	1.131 (0.582–2.200)	0.716		
**RASI**												
ACEI	[Reference] 1		[Reference] 1				[Reference] 1		[Reference] 1		[Reference] 1	
non-RASI	0.556 (0.348–0.891)	**0.015**	0.587 (0.361–0.957)	**0.033**	0.536 (0.333–0.864)	**0.010**	0.808 (0.479–1.364)	0.426	0.739 (0.423–1.291)	0.288	0.739 (0.433–1.261)	0.267
ARB	0.366 (0.167–0.801)	**0.012**	0.347 (0.156–0.773)	**0.010**	0.326 (0.147–0.724)	**0.006**	0.449 (0.189–1.065)	0.069	0.353 (0.140–0.892)	**0.028**	0.339 (0.135–0.850)	**0.021**
**Alpha blockers**												
No	[Reference] 1						[Reference] 1					
Yes	0.857 (0.395–1.857)	0.695					0.964 (0.387–2.401)	0.937				
**Beta blockers**												
No	[Reference] 1						[Reference] 1					
Yes	0.991 (0.654–1.502)	0.968					0.901 (0.553–1.468)	0.675				
**Calcium channel blockers**												
No	[Reference] 1						[Reference] 1					
Yes	1.124 (0.699–1.805)	0.630					1.084 (0.617–1.906)	0.779				
**Diuretics**												
No	[Reference] 1						[Reference] 1					
Yes	0.883 (0.565–1.380)	0.586					1.192 (0.720–1.973)	0.494				

*OS* Overall survival, *DFS* Disease-free survival, *ACEI* Angiotensin-converting enzyme inhibitors, *ARB* Angiotensin receptor blockers, *RASI* Renin-angiotensin system inhibitors, *PNI* Perineural invasion, *uHR* univariate hazard ratio, *mHR* Hazard ratio for the multivariate model with covariate preselection based on the univariate analysis, *mrHR* hazard ratio for the reduced multivariate model with covariate preselection based on the univariate analysis. *BMI* Body mass index, *CAD* Coronary artery disease, *DM* Diabetes mellitus, *CCI* Charlson Comorbidity Index, *RT* Radiotherapy, *CRT* Chemoradiotherapy, *AR* Anterior resection, *APR* Abdominoperineal resection, *Hartm* Hartmann’s procedure LNY- lymph node yield, *TRG* Tumor regression grade, *LVI* Lymphovascular invasion, *CT* Chemotherapy

## Discussion

Recently, the role of tRAS has been discussed in the pathogenesis and progression of some cancers. The mechanisms of the influence of tRAS on cancer progression may be diverse and can be associated with the effects on proliferation, migration, angiogenesis and immunosuppression [5]. The components of tRAS are present in cells of many cancers, including colorectal cancer and its microenvironment, such as tumor-associated macrophages, regulatory T-cells, or fibroblasts. Through the mechanism of AT1R activation, these cells induce immunosuppression in the tumor microenvironment and affect tumor progression and increase metastatic potential [5, 16]. Studies using animal models showed that this effect could be reduced by ARBs, which selectively block AT1R [16]. AT1R activation increases the expression of vascular endothelial growth factor (VEGF), which is the main factor responsible for angiogenesis [17]. It was also shown that high expression of the *AGTR1* gene encoding the AT1R protein correlated with poorer long-term colorectal cancer outcomes [18].

In addition, through its direct vasoconstrictive effect, AngII, which is the main component of RAS, reduces perfusion in the tumor and its microenvironment leading to hypoxia and acidosis. By enhancing the expression of proinflammatory cytokines, these factors result in cancer-promoting inflammation [5]. To balance the pathway activated by AT1R, RAS also has the so-called “protective arm”, including the angiotensin II type 2 receptor (AT2R), ACE2, Angiotensin (1–7), and the Mas receptor (MasR). Its activation produces the effect opposite to the activation of AT1R, including vasodilatory, anti-inflammatory and antiproliferative effects, which are achieved by reducing cytokine levels or inhibiting VEGF expression [7, 17, 19].

When considering the potential influence of RAS on the pathogenesis and the course of cancer, its interactions with KKS should also be considered. Kinins show pro-tumorigenic properties due to their ability to stimulate angiogenesis, cell proliferation and migration [20]. Kallikrein is the main enzyme causing kinin formation, while ACE is the main enzyme cleaving bradykinin (BK) into an inactive form [BK(1–7)]. Thus, the concentration of kinins in tissues depends on the local balance between these two enzymes [21]. Blocking ACE results in an increase in the concentration of BK and desArg9 BK, which is formed from BK under the influence of carboxypeptidases and is the most potent activator of the BK type 1 receptor (B1R). The expression of this receptor increases significantly under inflammatory conditions, whereas it is virtually undetectable under physiological conditions. Degradation of desArg9 BK into inactive metabolites is mediated by ACE2 [22].

The impact of RASIs on this complex mechanism of mutual relationships is poorly understood as regards colorectal cancer outcomes. A recent meta-analysis indicated a beneficial effect of RASIs on the survival of patients with gastrointestinal cancers. However, there are not many papers that assessed the impact of these drugs on colorectal cancer outcomes. In addition, most authors of the papers included in the meta-analysis analyzed the effect of both drug groups jointly (ACEIs/ARBs) [23]. The only meta-analysis which included only patients with colorectal cancer showed that RASIs could be associated with a reduced risk of colorectal cancer. However, no conclusions could be drawn in terms of the effect of these drugs on treatment outcomes [10]. Four studies on colorectal cancer patients, also including stage IV cancers, did not demonstrate the effect of ACEIs/ARBs on patient survival when the analyses without division into subgroups were performed [18, 24–26]. However, Ozawa et al. demonstrated their beneficial effect on recurrence-free survival in left-sided colorectal cancer and stage I subgroups [18]. In turn, Engineer et al. showed significantly better survival when RASIs were combined with a beta-blocker [24]. In a nested case–control study based on the national registry data, Cardwell et al. demonstrated a beneficial effect of ACEIs on cancer-specific mortality in colorectal cancer patients compared to non-users. However, no protective effect of ACEIs was reported after excluding the patients who had started using ACEIs in the year prior to death or when the analysis was restricted to users of any antihypertensive medication in the year prior to cancer diagnosis [27]. In contrast, Heinzerling et al. demonstrated that not using ACEIs was an unfavorable predictor of distant metastases in patients with stage II colorectal cancer [28]. The results of the study of the effect of ARBs on survival are also inconsistent. In our material, in patients treated with RASIs, we demonstrated a beneficial effect of ARBs on long-term survival. To the best of our knowledge, there have been no reports assessing the effect of RASI groups (i.e., ACEI vs. ARB) on long-term survival in rectal cancer patients after combined treatment. The results partially consistent with ours were presented by Cui et al. who showed significantly better OS and DFS in the users of ARBs or beta-blockers compared to those who did not use these drugs. However, the analysis covered colorectal cancer patients, including patients with stage IV disease [29]. Osumi et al. showed that in metastatic colorectal cancer, patients treated with bevacizumab who also used ARBs had significantly better OS and progression-free survival compared to ARB non-users [30]. However, Cardwell et al. found no effect of ARBs on colorectal cancer-specific mortality in the population-based study [27].

Only one paper assessed the effect of RASIs on the survival of rectal cancer patients only. However, both drug groups were evaluated jointly. Morris et al. showed that the use of ACEI/ARB significantly increased the rate of tumor pathological complete response (pCR) to preoperative RT. Those authors showed no effect of these drugs on OS, local recurrence-free survival, or metastasis-free survival; neither did they demonstrate the effect of pCR on survival [31]. In contrast, Rombouts et al. did not confirm the effect of ACEI/ARB on pCR. They showed a beneficial effect of beta-blockers in the multivariate analysis. However, they did not conduct the survival analysis [32]. In our study, we observed a higher percentage of positive responses to RT (TRG 0–1) in non-RASI users. We showed significantly worse OS in ACEI users compared to ARB and non-RASI users and worse DFS, which was close to the statistical significance level, in ACEI users compared to ARB users. In the multivariate Cox analysis, in addition to the influence of known risk factors such as age, ypN or PNI, the use of ACEIs was an unfavorable prognostic factor for OS, whereas ARBs showed a favorable effect on DFS. These results showed that tRAS could have a significant impact on the course of the disease, and its inhibition by different RASI groups may produce different effects. The potential mechanisms of this phenomenon are poorly understood, and hence further studies are warranted. They are most likely due to the different mechanisms of action of both RASI groups.

ARBs block the RAS more effectively than ACEIs because approximately 40% of AngII is formed in non-ACE pathways [8, 33]. In addition, while ARBs selectively block the ACE/AngII/AT1R proinflammatory pathway, they can simultaneously activate the AT2R/ACE2/Ang1-7/MasR anti-inflammatory pathway [4, 34]. Such diverse effects are not demonstrated by ACEIs, which may additionally exert adverse effects by blocking kinin degradation. Our results indicate that further studies are necessary to confirm whether the use of ARBs (instead of ACEIs) may lead to improved long-term oncological outcomes in rectal cancer patients. It is crucial since both groups of drugs have comparable efficacy in the treatment of cardiovascular disease. However, a lower risk of side effects is reported in the case of ARBs [9]. It seems that it is warranted to analyze ARBs and ACEIs separately in terms of their impact on long-term oncological outcomes because their different mechanisms of action may differently affect the course of the cancer disease.

The study has limitations typical of single-center and retrospective analyses. Data on comorbidities and drug use were collected from the records of consultant internal physicians and anesthesiologists before surgery. It was not possible to assess the duration of drug use. The smaller size of the group of ARB users is due to the fact that ARBs are less commonly used compared to ACEIs. As we showed in an additional analysis, it was not associated with the socioeconomic status of our patients. However, the level of education was the only parameter available to assess the socioeconomic status of the study group due to the specificity of the Upper Silesian Conurbation where our Institute is located and the restrictions resulting from the law regulations (Additional file 4).

## Conclusions

The use of ARBs, instead of ACEIs, may improve the long-term outcome of the combined treatment of rectal cancer patients with associated hypertension.

## Supplementary Information

**Additional file 1.** Diagram showing the formation of the study group.

**Additional file 2.** Cox proportional-hazards model.

**Additional file 3.** Kaplan-Meier Survival Analysis.

**Additional file 4.** Socioeconomic analysis.

**Additional file 5.** Dataset.

## Data Availability

The dataset supporting the conclusions of this article is included in the article [see Additional file 5].

## Abbreviations

BMI: Body mass index
ACE: Angiotensin-converting enzyme
AngII: Angiotensin II
Ang(1–7): Angiotensin (1–7)
RAS: Renin-angiotensin system
tRAS: Tissue renin-angiotensin system
RASI: Renin-angiotensin system inhibitors
ARB: Angiotensin receptor blockers
ACEI: Angiotensin-converting enzyme inhibitors
AT1R: Angiotensin II type I receptor
AT2R: Angiotensin II type II receptor
OS: Overall survival
DFS: Disease-free survival
KKS: Kallikrein-kinin system
RT: Radiotherapy
CRT: Chemoradiotherapy
CCI: Charlson Comorbidity Index
CKD: Chronic kidney disease
DM: Diabetes mellitus
CAD: Coronary artery disease
LVI: Lymphovascular invasion
PNI: Perineural invasion
TRG: Tumor regression grade
pCR: Pathological complete response
LNY: Lymph node yield
IQR: Interquartile range
OR: Odds ratio
HR: Hazard ratio
CI: Confidence interval
B1R: Bradykinin type 1 receptor
MasR: Mas receptor
BK: Bradykinin
