# Supplementary material for: Impact of renin-angiotensin system inhibitors on the survival of patients with rectal cancer
Source: BMC Cancer. 2022 Jul 25;22:815. doi: 10.1186/s12885-022-09919-0 (PMC9316329; doi:10.1186/s12885-022-09919-0)
Supplement: Supplementary file 1 — Additional file 1. Diagram showing the formation of the study group. [file 12885_2022_9919_MOESM1_ESM.docx]

Diagram showing the formation of the study group

(-)

(-)

(-)

(-)

Radical resection after RT or CRT with associated AH

THE STUDY GROUP n= 242

Postoperative death (n=9)

Patients with no AH (n=347)

Radical resection after RT or CRT

(N=598)

No neoadjuvant treatment

(N=177)

AR (n=456)

APR (n=267)

Hartmann's procedure (n=52)

(N=775)

Local resection (n=28)

Radical resection (n=803)

Patients with dissemination (M1) n=103

Non-radical procedures R1/R2, including CRM+ (n=15)

Patients who underwent rectal cancer surgery between 2008 and 2016 (n=921)

CRM- circumferential radial margin, AR- anterior rectal resection, APR- abdominoperineal resection, AH- arterial hypertension, RT- radiotherapy, CRT- chemoradiotherapy
