# Supplementary material for: Impact of renin-angiotensin system inhibitors on the survival of patients with rectal cancer
Source: BMC Cancer. 2022 Jul 25;22:815. doi: 10.1186/s12885-022-09919-0 (PMC9316329; doi:10.1186/s12885-022-09919-0)
Supplement: Supplementary file 3 — Additional file 3. Kaplan-Meier Survival Analysis. [file 12885_2022_9919_MOESM3_ESM.docx]

**Additional file 3. Kaplan-Meier Survival Analysis:**

comparison of patient groups ARB and ACEI vs non-RASI


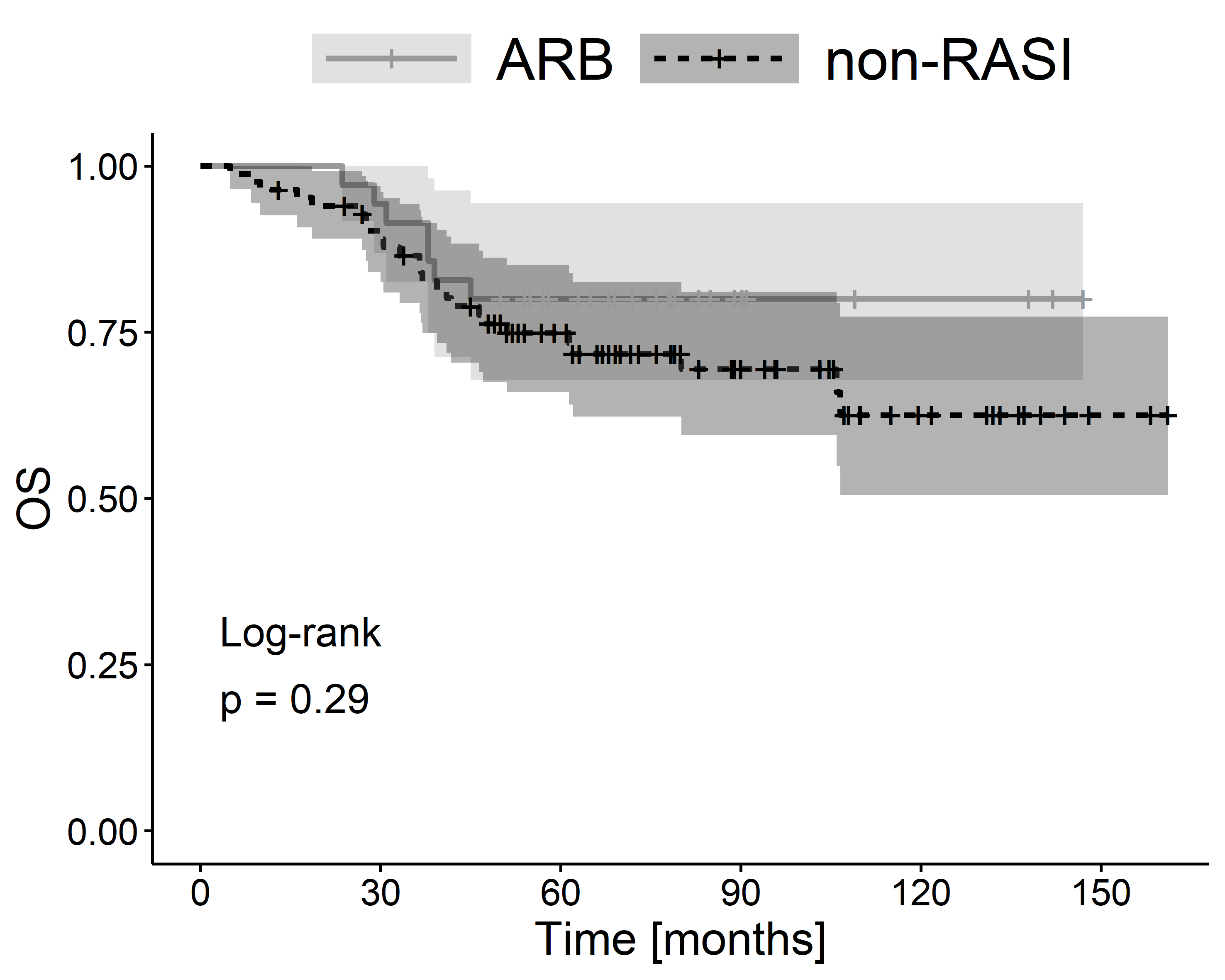


**Additional file 3A.** The Kaplan–Meier plots of overall survival (OS) for comparison of patient groups ARB vs non-RASI.


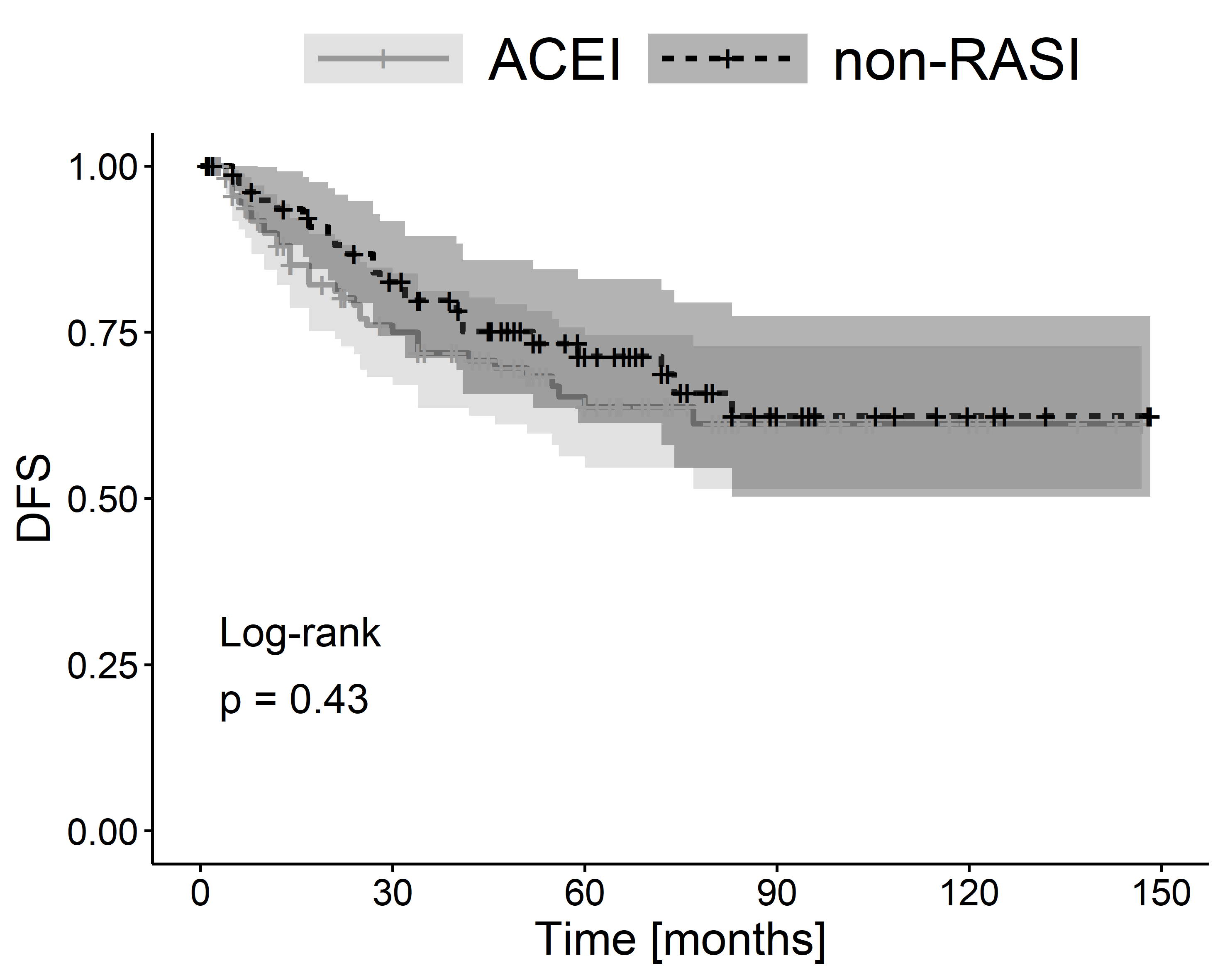

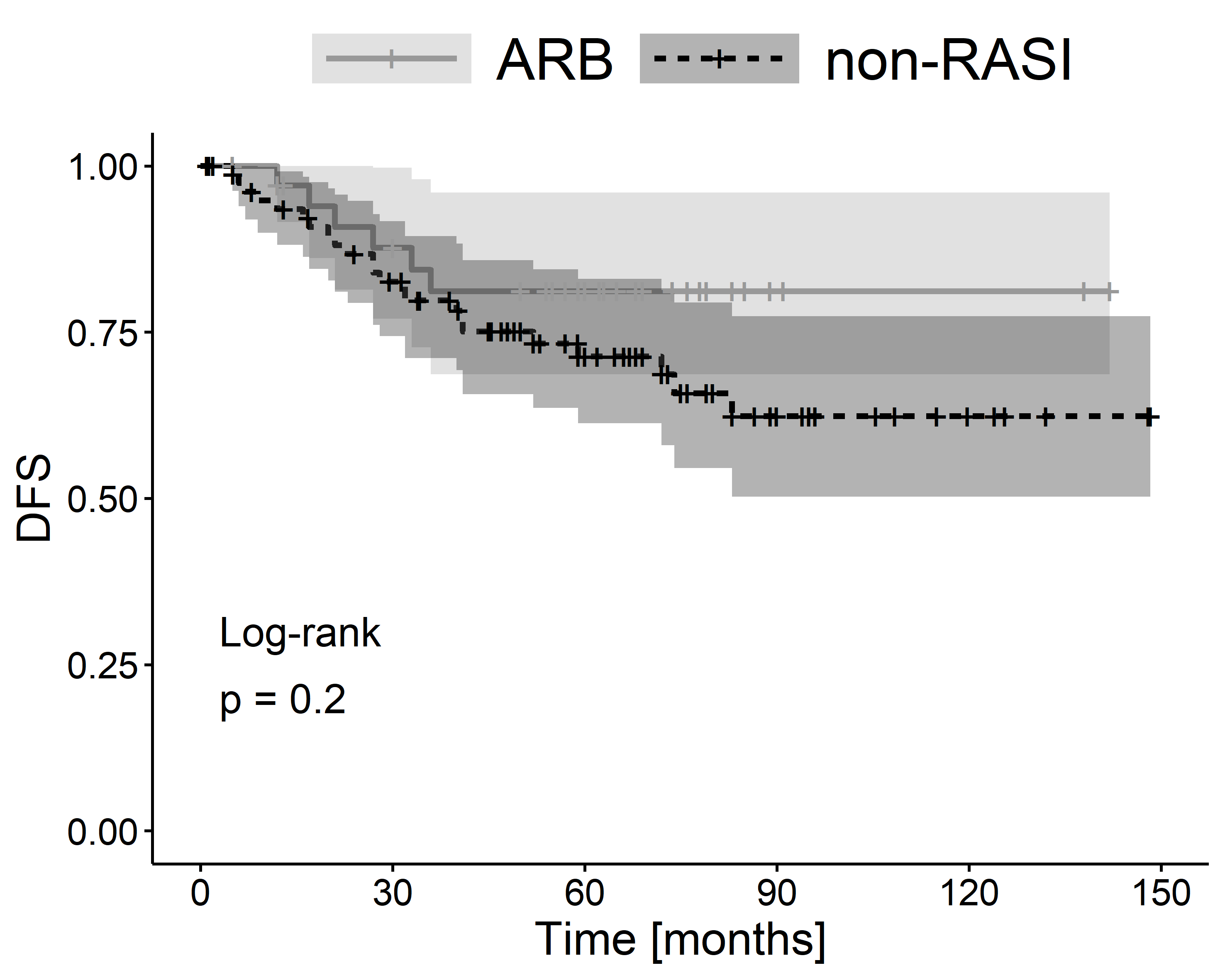


**Additional file 3B.** The Kaplan–Meier plots of disease free survival (DFS) for comparison of patient groups ACEI vs non-RASI and ARB vs non-RASI.
