# Supplementary material for: Impact of renin-angiotensin system inhibitors on the survival of patients with rectal cancer
Source: BMC Cancer. 2022 Jul 25;22:815. doi: 10.1186/s12885-022-09919-0 (PMC9316329; doi:10.1186/s12885-022-09919-0)
Supplement: Supplementary file 4 — Additional file 4. Socioeconomic analysis. [file 12885_2022_9919_MOESM4_ESM.docx]

Assessment of the effect of the socioeconomic status and drug prices on the frequency of use of both RASI groups during the analyzed period

The comparison was conducted based on the two drugs that were most frequently used in our study group, i.e. ramipril (5mg) and losartan (50mg). Below we present the real average prices of the drugs in PLN in 2008, 2012 and 2016 (with consideration given to reimbursement).

|  | 2008 | 2012 | 2016 |
| --- | --- | --- | --- |
| ARB  (Losartan 50mg, 30 tablets) | 13.20 | 11.60 | 6.27 |
| ACEI  (Ramipril 5mg, 30 tablets) | 4.35 | 6.83 | 6.40 |
| The difference between the prices of ARB and ACEI (in PLN) | 8.86 | 4.77 | -0.13 |

Despite the fact that at the beginning and in the middle period of the analysis the monthly cost of ARB therapy was higher than that of ACEI, these drugs were in the same price group and the absolute value differences were not high, considering the average earnings in Poland at that time. This was due to reimbursement of the drugs by the State. Below are the average earnings in PLN in 2008, 2012 and 2016.

| 2008 | 2943.88 |
| --- | --- |
| 2012 | 3521.67 |
| 2016 | 4047.21 |

Thus, the price differences between ARB and ACEI in 2008, 2012, and 2016 were 0.3%, 0.1%, and 0% of average earnings, respectively.

Next, we attempted to analyze socioeconomic factors. The factor that would best determine the wealth status of patients would be the amount of income. However, according to the Polish law, we do not have access to such data. The only factors available to us that could indirectly indicate the socioeconomic status are education and the place of residence (village/town/city size, number of inhabitants). We included such information in the Dataset. However, most patients treated in our center live in the Upper Silesian Conurbation, in which, due to its specificity, the place of residence does not indicate the financial status or the access to medical care. Due to the long-standing trend of some wealthy city dwellers to move to nearby small towns and villages, any attempt to correlate the place of residence with the socioeconomic status would be inadequate and incorrect. Therefore, education is the only available parameter. We performed the analysis of OS depending on education. Statistical significance was not obtained in the log-rank test (p= 0.62).


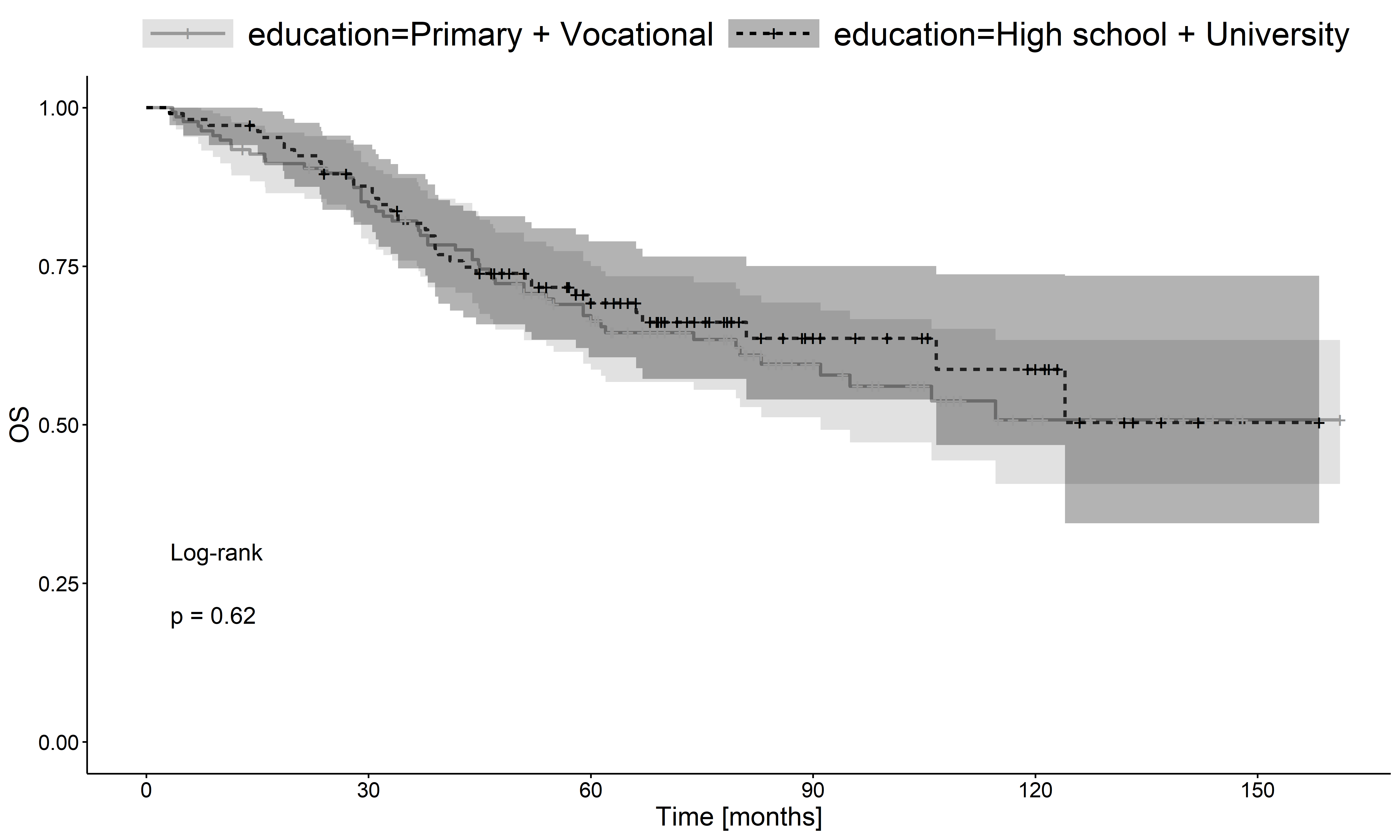


The univariate Cox analysis did not show a significant effect of education on OS.


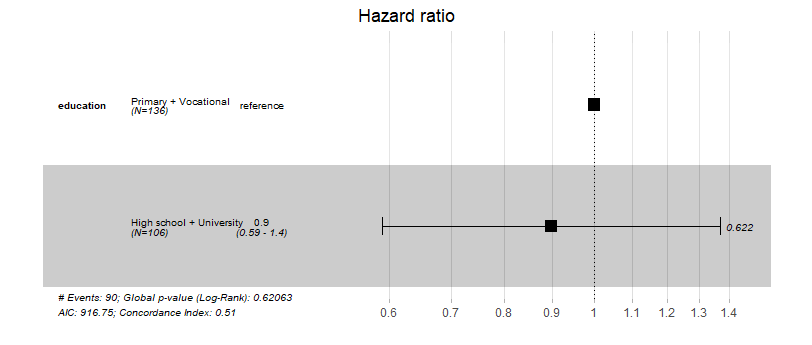


Additionally, we found no differences in the frequency of use of specific groups of drugs depending on education (Fisher exact test; p= 0.936).

|  | non-RASi | ARB | ACEI |
| --- | --- | --- | --- |
| Primary + Vocational | 46 | 20 | 70 |
| High school + University | 38 | 15 | 53 |

Next, we examined whether there were differences in the frequency of medication use in the following years of the analysis by establishing 3 intervals.

|  | ARB (n) | ACEI (n) | non-RASi (n) |
| --- | --- | --- | --- |
| 2008-2010 | 3 | 28 | 20 |
| 2011-2013 | 10 | 43 | 28 |
| 2014-2016 | 22 | 52 | 36 |

We found no significant differences for the above 3 drug groups (p= 0.19) or for ARB vs. ACEI (p= 0.07); (Fisher exact test).

Conclusions

Based on the above additional analysis, we concluded that there was no basis for the opinion that the cost of the drugs was the cause of the difference in the size of the groups on ARB and RASI.

Additionally, we found no significant differences in the frequency of use of the analyzed drug groups in different time intervals covering the analyzed period (2008-2010; 2011-2013; 2014-2016).

The level of education was the only parameter available to assess the socioeconomic status of the study group. The analysis of this parameter showed no significant effect on long-term survival in the log-rank test or the univariate Cox analysis.
